# Career Outcomes and Perceptions of Alumni who completed Postgraduate Education in Sciences in Gastroenterology: insights from a Brazilian Single-Center Survey

**DOI:** 10.1590/0102-672020260000021e1950

**Published:** 2026-08-17

**Authors:** Gilton Marques FONSECA, Rafaela Maria Zacchi MARTUCCI, José JUKEMURA, Claudia Pinto Marques Souza OLIVEIRA, Alberto Queiroz FARIAS, Ulysses RIBEIRO, Paulo HERMAN

**Affiliations:** 1 Universidade de São Paulo, Department of Gastroenterology, School of Medicine – São Paulo (SP), Brazil.

**Keywords:** Education, Graduate, Internship and Residency, Surgical Procedures, Operative, Colorectal Surgery, Digestive System Surgical Procedures, Educação de Pós-Graduação, Internato e Residência, Procedimentos Cirúrgicos Operatórios, Cirurgia Colorretal, Procedimentos Cirúrgicos do Sistema Digestório

## Abstract

**Background:**

Postgraduate education is essential for training highly qualified health professionals; however, there is a lack of studies in Brazil evaluating career outcomes of gastroenterology alumni.

**Aims:**

To analyze the sociodemographic profile, academic background, career outcomes, and perceived impact of postgraduate training in Sciences, more specifically in Gastroenterology, comparing physicians and non-physicians, as well as physicians with surgical versus clinical training.

**Methods:**

A cross-sectional study was conducted among alumni who completed master’s and doctoral degrees from 2012 to 2024 at Universidade de São Paulo School of Medicine. A 47-item questionnaire collected data on demographics, training, professional practice, academic productivity, and program impact. Statistical comparisons were performed using ꭓ^2^ or Fisher’s exact tests and the Mann-Whitney U test (p<0.05).

**Results:**

Of the 332 eligible alumni, 172 completed the questionnaire (51.8%). The mean age was 43 years; 53.5% were men, and 69.8% were physicians. Postgraduate training was considered fundamental by 51.2% and highly impactful by 37.2, and 74.4% would certainly pursue it again. Physicians were older, had higher scientific output, and more frequently held academic and leadership positions. Non-physicians were predominantly women, received a higher number of scholarships, and more frequently reported positive career changes. Among physicians, those with surgical training had higher income, more academic titles, and were more often based in São Paulo, whereas those with clinical training reported more professional opportunities.

**Conclusions:**

Postgraduate training in Sciences in the field of Gastroenterology, had a relevant positive impact on academic and professional development, with differences according to baseline training and professional profile.

## ARTICLE HIGHLIGHTS

Postgraduate education in Brazil was formally established in the 1960s and has become essential for scientific and technological development. In 2011, Universidade de São Paulo School of Medicine merged the Gastrointestinal Surgery and Clinical Gastroenterology programs into an interdisciplinary postgraduate program in Sciences in Gastroenterology for physicians and other health professionals. Despite the relevance of postgraduate training, studies evaluating alumni outcomes in gastroenterology remain scarce in Brazil. This study assessed the academic and professional trajectories of alumni, comparing career outcomes and perceived impact between physicians and non-physicians.

## Summary

**CENTRAL MESSAGE:**

Postgraduate education has a relatively recent history in Brazil. In 2011, the Gastrointestinal Surgery and Clinical Gastroenterology programs were officially merged to form the Postgraduate Program in Sciences in Gastroenterology, offering both master’s and doctoral degrees. Despite its importance, systematic evaluation of alumni outcomes remains scarce in Brazil, particularly in the field of gastrointestinal surgery and gastroenterology. Most of the data available derive from international experiences or broader surveys in health sciences. Identifying the sociodemographic profile, academic background, career paths, and perceived impact of postgraduate education is crucial for guiding institutional strategies and contributing to the evaluation metrics by the Coordination for the Improvement of Higher Education Personnel.

**PERSPECTIVES:**

This survey of alumni from a postgraduate program in Sciences in Gastroenterology demonstrates that postgraduate studies have a strong impact on both personal and professional development. Most alumni reported career and academic growth, and the majority would certainly enroll again. Differences between physicians and non-physicians, and between surgical and clinical training, highlight the diverse ways postgraduate education shapes careers. At the same time, limited research funding and the concentration of professionals in large urban centers remain persistent challenges. These findings reinforce the pivotal role of postgraduate education in advancing health sciences while pointing to opportunities for greater equity and improved regional distribution of qualified professionals.

## VISUAL ABSTRACT

**Figure:**
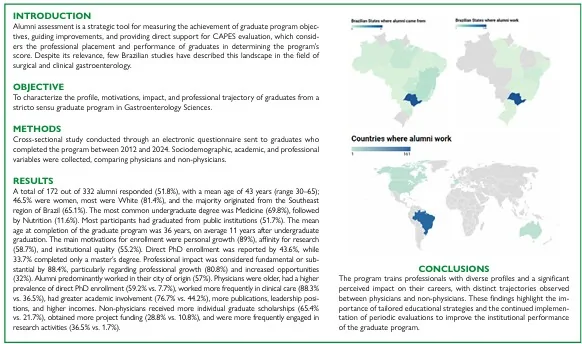

## RESUMO

**Racional:**

A pós-graduação é fundamental para a formação de profissionais qualificados em saúde, porém há escassez de estudos no Brasil que avaliem os desfechos de egressos na área de gastroenterologia.

**Objetivos:**

Analisar o perfil sociodemográfico, formação acadêmica, desfechos de carreira e a percepção do impacto da pós-graduação em Ciências em Gastroenterologia, comparando médicos e não médicos, bem como médicos com formação cirúrgica versus clínica.

**Métodos:**

Estudo transversal com egressos de mestrado e doutorado formados entre 2012 e 2024 na Faculdade de Medicina da Universidade de São Paulo. Aplicou-se questionário com 47 itens abordando dados demográficos, formação, atuação profissional, produção acadêmica e impacto do programa. As comparações estatísticas utilizaram testes do ꭓ^2^ ou exato de Fisher e o teste de Mann-Whitney (p<0,05).

**Resultados:**

Dos 332 egressos elegíveis, 172 responderam ao questionário (51,8%). A idade média foi de 43 anos; 53,5% eram homens e 69,8% médicos. A pós-graduação foi considerada fundamental por 51,2% e altamente impactante por 37,2, e 74,4% certamente a fariam novamente. Médicos apresentaram maior idade, maior produção científica e maior frequência de cargos acadêmicos e de liderança. Não médicos foram predominantemente mulheres, receberam mais bolsas e relataram com maior frequência mudança positiva de carreira. Entre os médicos, aqueles com formação cirúrgica tiveram maior renda, mais títulos acadêmicos e maior concentração em São Paulo, enquanto os de formação clínica relataram mais oportunidades profissionais.

**Conclusões:**

A pós-graduação em Ciências em Gastroenterologia exerceu impacto positivo relevante no desenvolvimento acadêmico e profissional, com diferenças conforme a formação de base e o perfil profissional.

## INTRODUCTION

 Postgraduate education has a relatively recent history in Brazil. Although initiatives in advanced training can be traced back to the early 20th century, the formal postgraduate system was only structured in the mid-1960s, following the recommendations of the Sucupira Report (1965)^10^. This document set the foundations for master’s and doctoral postgraduate programs, aligning Brazil with international standards for master’s and doctoral degrees. Since then, postgraduate education has expanded substantially, becoming a central component of scientific and technological development in the country^1,2,13^. 

 The Coordination for the Improvement of Higher Education Personnel (CAPES) was created to promote postgraduate education and evaluate the performance of programs nationwide. In its assessment system, the trajectory of alumni has become an increasingly relevant metric, as it reflects not only the academic output of programs but also their ability to train professionals who contribute to teaching, research, healthcare, and innovation^2,14^. 

 In recent years, several initiatives have strengthened the postgraduate programs at Universidade de São Paulo School of Medicine (FMUSP). In 2011, the Gastrointestinal Surgery and Clinical Gastroenterology programs were officially merged to form the Postgraduate Program in Sciences in Gastroenterology, offering both master’s and doctoral degrees. The present study focuses on alumni who completed their studies after this integration, ensuring that the analysis reflects the program’s consolidated structure. Importantly, the program admits not only physicians but also professionals from related health fields, such as nutrition, pharmacy, psychology, and others, broadening its scope and reinforcing the interdisciplinary impact of postgraduate training^5,7,8^. 

 Despite its importance, systematic evaluation of alumni outcomes remains scarce in Brazil, particularly in the field of gastrointestinal surgery and gastroenterology. Most of the data available derive from international experiences or broader surveys in health sciences. Identifying the sociodemographic profile, academic background, career paths, and perceived impact of postgraduate education is crucial for guiding institutional strategies and contributing to CAPES evaluation metrics^3,4^. 

 This study aimed to evaluate the professional and academic trajectories of alumni from a postgraduate program in Sciences in Gastroenterology, analyzing their sociodemographic profiles, career outcomes, and perceived impact. We also compared physicians with non-physicians to better understand potential differences in career development and professional opportunities after completing postgraduate education. 

## METHODS

 This cross-sectional survey included alumni of the Postgraduate Program in Sciences in Gastroenterology at FMUSP. Eligible participants were individuals who completed master’s or doctoral studies between 2012 and 2024. Individuals who did not complete the questionnaire or submitted incomplete questionnaires were excluded. 

 Alumni were invited through an electronic link distributed via e-mail and instant messaging applications. The survey consisted of 47 questions and was designed to be completed in approximately seven minutes. Mandatory and conditional branching items were included, and multiple responses were allowed when applicable. For alumni living abroad, technical issues prevented access to REDCap; therefore, Google Forms or a PDF version of the questionnaire was provided. Up to four weekly reminders were sent, and non-respondents were followed individually. No incentives were offered. 

 The questionnaire assessed sociodemographic characteristics, undergraduate and postgraduate education, scholarships and funding, professional trajectory, academic and research activities, and perceptions regarding the impact of postgraduate studies. 

 Categorical variables were summarized as frequencies and percentages, whereas continuous variables were expressed as means and standard deviations or medians and interquartile ranges, depending on the distribution. Comparisons between groups were performed using the chi-square test or Fisher’s exact test for categorical variables, and the Mann-Whitney U test for continuous variables. A p<0.05 was considered statistically significant. Statistical analyses were conducted using IBM SPSS Statistics, version 26.0 (IBM Corp., Armonk, NY, USA). 

 The study was conducted according to the Declaration of Helsinki and approved by the Institutional Ethics Committee (protocol number 79812624.9.0000.0068). All participants provided informed consent. 

## RESULTS

 A total of 332 alumni who had completed postgraduate studies in Sciences in Gastroenterology from January 2012 to December 2024 were identified and contacted. Of these, 174 responses were received, and two were excluded due to incompleteness, resulting in 172 valid questionnaires, a response rate of 51.8%. 

**Figure 1:**
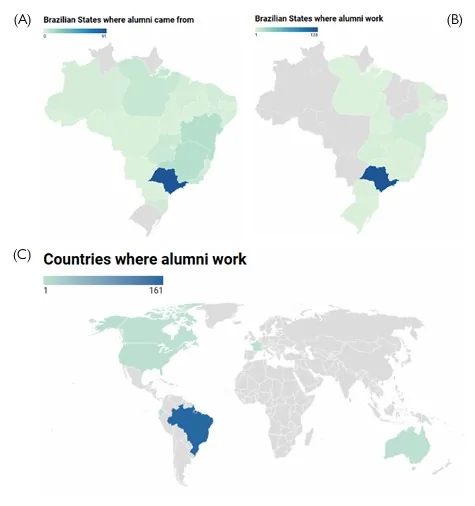
Maps depicting: (A) Brazilian States where alumni* came from; (B) Brazilian States where alumni* currently work; and (C) Countries where alumni* work. *Alumni who completed postgraduate studies in Sciences in Gastroenterology at Universidade de São Paulo School of Medicine, from January 2012 to December 2024. Source: Personal archive.

 The mean age of respondents was 43 years (standard deviation (SD) 6.9; range 30-65), with a median of 42 years. Slightly more than half were male (92; 53.5%), and most selfidentified as Caucasian (140; 81.4%). Alumni came from every region of Brazil, with the Southeast region being the most represented (112; 65.1%). Five (2.9%) respondents were from outside Brazil (Figure 1A). 

 Regarding academic background, most respondents held a medical degree (120; 69.8%), followed by nutrition (20; 11.6%), pharmacy (7; 4.1%), psychology (6; 3.5%), and other health disciplines. About half graduated from public universities (89; 51.7%), including 26 (15.1%) from FMUSP. With respect to their postgraduate trajectories, 75 (43.6%) had a direct-entry doctoral degree, 58 (33.7%) completed only a master’s degree, and 30 (17.4%) completed both degrees. The mean interval between undergraduate and postgraduate completion was 12 years (range 4–32), and the mean age at postgraduate completion was 36 years (range 25–56). 

 Scholarships during postgraduate studies were reported by 60 (34.9%) alumni, mostly funded by CAPES (44; 73.4%), FAPESP (São Paulo Research Foundation) (11; 18.3%), and CNPq (National Council for Scientific and Technological Development) (5; 8.3%). Project funding was less common (28; 16.3%), predominantly from FAPESP (23; 85.2%). Motivations for pursuing postgraduate studies included professional growth (153; 89%), personal growth (109; 63.4%), affinity for research (101; 58.7%), and institutional quality (95; 55.2%). 

 A substantial portion perceived postgraduate studies as having a fundamental impact on their professional life (88; 51.2%), with another 64 (37.2%) reporting a great but not decisive impact. Personal and professional growth were commonly attributed benefits (77.9% and 80.8%, respectively), and 128 (74.4%) would certainly pursue postgraduate studies again. 

 Regarding professional practice, 125 (72.7%) work primarily in healthcare, followed by research (21; 12.2%), teaching (17; 9.9%), and management (7; 4.1%). Most work in public and private institutions (68; 39.5%) or exclusively in the private healthcare sector (61; 35.5%). Academic activity was maintained by 115 (66.9%), and 96 (55.8%) engaged in research. The number of publications ranged widely: 62 (36%) reported 1-5 papers, 57 (33.1%) reported 6-20 papers, six had no publications, and six (3.5%) had more than 150 papers. Leadership roles were reported by 72 (41.9%), and 20 (11.6%) served on editorial boards. The current work location was heavily concentrated in São Paulo city (118; 68.6%), with 98 (57%) working in their city of origin (Figure 1B-C). Table 1 summarizes the characteristics of the population. 

**Table 1:** Characteristics of alumni who completed postgraduate studies in Sciences in Gastroenterology at Universidade de São Paulo School of Medicine, from January 2012 to December 2024.

Variable	Characteristic	n (%)
Sex	Male	92 (53.5)
	Female	80 (46.5)
Current age (years)	Mean (SD)	43 (6.9)
	Median (min-max)	42 (30-65)
Ethnicity	Caucasian	140 (81.4)
	Asian	11 (6.4)
	Black/brown	17 (9.9)
	Indigenous	1 (0.6)
	Prefer not to answer	3 (1.7)
Region of origin	Southeast	112 (65.1)
	Northeast	26 (15.1)
	South	11 (6.4)
	North	11 (6.4)
	Midwest	7 (4.1)
	Foreigner	5 (2.9)
Came from the São Paulo capital city	Yes	64 (37.2)
	No	108 (62.8)
Graduation	Medicine	120 (69.8)
	Nutrition	20 (11.6)
	Pharmacy	7 (4.1)
	Psychology	6 (3.5)
	Biology	5 (2.9)
	Biomedicine	5 (2.9)
	Nursing	4 (2.3)
	Physiotherapy	3 (1.7)
	Physical education	1 (0.6)
	Gerontology	1 (0.6)
Graduated from a	Public institution	89 (51.7)
	Private institution	83 (48.3)
Postgraduation level	Doctorate (direct entry)	89 (51.7)
	Master’s degree	58 (33.7)
	Master’s degree and doctorate	39 (22.7)
Age at postgraduation completion (years)	Mean (SD)	36 (5.8)
	Median (min-max)	36 (25-56)
Time between graduation and postgraduation completion (years)	Mean (SD)	12 (5.6)
	Median (min-max)	12 (4-32)
Postgraduate scholarship	Yes	60 (34.9)
	No	112 (65.1)
Research project funding	Yes	28 (16.3)
	No	144 (83.7)
Motivations to pursue postgraduate studies	Personal growth	109 (63.4)
	Professional growth	153 (89.0)
	Increase income levels	25 (14.5)
	Obtain a postgraduate scholarship	7 (4.1)
	Affinity for the research field	101 (58.7)
	Facilitate a change of professional area	2 (1.2)
	Quality of the institution’s education	95 (55.2)
	Good reputation of the faculty	65 (37.8)
	Become a researcher	55 (32.0)
	Become a teacher/professor	68 (39.5)
	Pass public exams	16 (9.3)
	Increase opportunities in the job market	49 (28.5)
After completing postgraduate studies, which aspects were positively impacted?	Personal growth	134 (77.9)
	Professional growth	139 (80.8)
	Increased income levels	45 (26.2)
	Changed professional area	11 (6.4)
	Became a researcher	33 (19.2)
	Became a teacher/professor	44 (25.6)
	Passed public exams	18 (10.5)
	Had new job opportunities	55 (32.0)
Postdoctoral studies	Yes	27 (15.7)
	No	145 (84.3)
Academic title (Associate Professor)	Yes	9 (5.2)
	No	163 (94.8)
Workplace location	Southeast of Brazil	136 (79.1)
	Northeast of Brazil	13 (7.6)
	South of Brazil	6 (3.5)
	Midwest of Brazil	5 (2.9)
	North of Brazil	2 (1.2)
	Foreigner	10 (5.8)
Would you pursue postgraduate studies again?	Definitely yes	128 (74.4)
	Likely	32 (18.6)
	Maybe	7 (4.1)
	Unlikely	5 (2.9)
Is your workplace in São Paulo, the capital	Yes	118 (68.6)
	No	54 (31.4)
Work in their city of origin	Yes	98 (57.0)
	No	74 (43.0)
Predominant field of work	Healthcare/Clinical practice	125 (72.7)
	Research	21 (12.2)
	Teaching	17 (9.9)
	Management	7 (4.1)
	Unemployed	2 (1.2)
Institution of work	Public and private	68 (39.5)
	Only private	61 (35.5)
	Only public	40 (23.3)
	Not applicable	3 (1.7)
Academic activities	Yes	115 (66.9)
	No	57 (33.1)
Research activities	Yes	96 (55.8)
	No	76 (44.2)
Number of papers published	None	6 (3.5)
	1 to 5	62 (36.0)
	6 to 20	57 (33.1)
	21 to 50	27 (15.7)
	51 to 100	14 (8.1)
	More than 100	6 (3.5)
Editorial board member	Yes	20 (11.6)
	No	152 (88.4)
Leadership positions	Yes	72 (41.9)
	No	100 (58.1)
Salary range	Up to BRL 3,000	4 (2.3)
	BRL 3,001 to BRL 10,000	26 (15.1)
	BRL 10,001 to BRL 20,000	33 (19.2)
	More than BRL 20,000	84 (48.8)
	Prefer not to disclose	25 (14.5)
Digital platforms	Open Researcher and Contributor ID (ORCID)	142 (82.6)
	LinkedIn	112 (65.1)
	Research Gate	79 (45.9)
	Google Scholar	61 (35.5)
	None	14 (8.1)

 Comparisons between physicians and non-physicians revealed significant differences (Table 2). Physicians were older, graduated more often from public universities, and were more likely to have a direct-entry doctoral degree. They also reported greater academic involvement, higher publication output, more leadership roles, and higher income levels. Non-physicians were predominantly women, more often based in São Paulo city, and more likely to receive scholarships or project funding. Career change as a positive impact was significantly more common among non-physicians (15.4 vs. 2.5%, p=0.003). 

**Table 2:** Univariate analysis comparing physicians and non-physicians among alumni who completed postgraduate studies in Sciences in Gastroenterology at Universidade de São Paulo School of Medicine, from January 2012 to December 2024.

Variable		n (%)		p-value
		Non-Physicians	Physicians	
Sex				
	Male	7 (13.5)	85 (70.8)	<0.001
	Female	45 (86.5)	35 (29.2)	
Ethnicity				
	Caucasian	43 (83)	97 (81)	0.539
	Asian	5 (10)	6 (05)	
	Black/brown	4 (08)	13 (11)	
	Indigenous	0 (00)	1 (01)	
	Prefer not to answer	0 (00)	3 (03)	
Came from São Paulo, the capital				
	Yes	30 (58)	34 (28)	<0.001
	No	22 (42)	86 (72)	
Graduated from a				
	Public institution	12 (23)	77 (64)	<0.001
	Private institution	40 (77)	43 (36)	
Postgraduation level				
	Doctorate (direct entry)	4 (08)	71 (59)	<0.001
	Master’s degree	32 (62)	26 (22)	
	Master’s degree and doctorate	16 (31)	28 (19)	
Postgraduation scholarship				
	Yes	34 (65)	26 (22)	<0.001
	No	18 (35)	94 (78)	
Research project funding				
	Yes	15 (29)	13 (11)	0.004
	No	37 (71)	107 (89)	
Motivations to pursue postgraduate studies				
	Personal growth	31 (60)	78 (65)	0.605
	Professional growth	45 (87)	108 (90)	0.597
	Increase income levels	11 (21)	14 (12)	0.156
	Obtain a postgraduate scholarship	4 (08)	3 (03)	0.201
	Affinity for the research field	36 (69)	65 (54)	0.091
	Change of professional area	0 (00)	2 (02)	1
	Quality of the institution’s education	30 (58)	65 (54)	0.739
	Good reputation of the faculty	` (33)	48 (40)	0.396
	Become a researcher	20 (38)	35 (29)	0.286
	Become a teacher/professor	17 (33)	51 (43)	0.24
	Pass public exams	5 (10)	11 (09)	1
	Opportunities in the job market	18 (35)	31 (26)	0.272
After completing postgraduate studies, which aspects were positively impacted?				
	Personal growth	40 (77)	94 (78)	0.843
	Professional growth	39 (75)	100 (83)	0.212
	Increased income levels	17 (33)	28 (23)	0.257
	Changed professional area	8 (15)	3 (03)	0.003
	Became a researcher	10 (19)	23 (19)	1
	Became a teacher/professor	8 (15)	36 (30)	0.057
	Passed public exams	1 (02)	17 (14)	0.014
	Had new job opportunities	20 (38)	35 (29)	0.286
Postdoctoral studies				
	Yes	7 (13)	20 (17)	0.656
	No	45 (87)	100 (83)	
Academic title (Associate Professor)				
	Yes	0 (00)	9 (08)	0.059
	No	52 (100)	111 (93)	
Is your workplace in São Paulo, the capital?				
	Yes	43 (83)	75 (63)	0.012
	No	9 (17)	45 (38)	
Work in their city of origin				
	Yes	34 (65)	64 (53)	0.180
	No	18 (35)	56 (47)	
Predominant field of work				
	Healthcare/Clinical practice	19 (37)	106 (88)	<0.001
	Research	19 (37)	2 (02)	
	Teaching	6 (12)	11 (09)	
	Management	6 (12)	1 (01)	
	Unemployed	2 (04)	0 (00)	
Institution of work				
	Public and private	8 (15)	60 (50)	<0.001
	Only private	28 (54)	33 (28)	
	Only public	14 (27)	26 (22)	
	Not applicable	2 (04)	1 (01)	
Academic activities				
	Yes	23 (44)	92 (77)	<0.001
	No	29 (56)	28 (23)	
Research activities				
	Yes	25 (48)	71 (59)	0.186
	No	27 (52)	49 (41)	
Number of papers published				
	None	5 (10)	1 (01)	0.002
	1 to 5	29 (56)	33 (28)	
	6 to 20	11 (21)	46 (38)	
	21 to 50	5 (10)	22 (18)	
	51 to 100	2 (04)	12 (10)	
	More than 100	0 (00)	6 (05)	
Editorial board member				
	Yes	2 (04)	18 (15)	0.039
	No	50 (96)	102 (85)	
Leadership positions				
	Yes	15 (29)	57 (48)	0.029
	No	37 (71)	63 (53)	
Salary range				
	Up to BRL 3.000	4 (08)	0 (00)	<0.001
	BRL 3.001 to BRL 10.000	21 (40)	5 (04)	
	BRL 10.001 to BRL 20.000	20 (38)	13 (11)	
	More than BRL 20.000	3 (06)	81 (68)	
	Prefer not to disclose	4 (08)	21 (18)	
Digital platforms				
	ORCID	40 (77)	40 (77)	0.273
	LinkedIn	42 (81)	70 (58)	
	Research Gate	23 (44)	56 (47)	
	Google Scholar	17 (33)	44 (37)	
	None	4 (08)	10 (08)	

 Among physicians, those with surgical training differed from those with clinical training (Table 3). Surgeons were more frequently male, more likely to work in São Paulo, and held more senior academic titles. Clinicians more often reported new job opportunities after completing postgraduate studies. Surgeons had higher income levels and were more frequently indexed in Google Scholar. 

**Table 3:** Univariate analysis comparing physicians with surgical training and those with clinical training among alumni who completed postgraduate studies in Sciences in Gastroenterology at Universidade de São Paulo School of Medicine, from January 2012 to December 2024.

Variable		n (%)		p-value
		Clinical training	Surgical training	
Sex				
	Male	28 (48)	57 (92)	<0.001
	Female	30 (52)	5 (08)	
Ethnicity				
	Caucasian	42 (72)	55 (89)	0.087
	Asian	5 (09)	1 (02)	
	Black/brown	10 (17)	3 (05)	
	Indigenous	0 (00)	1 (02)	
	Prefer not to answer	1 (02)	2 (03)	
Came from São Paulo, the capital				
	Yes	11 (19)	23 (37)	0.042
	No	47 (81)	39 (63)	
Graduated from a				
	Public institution	38 (66)	39 (63)	0.850
	Private institution	20 (34)	23 (37)	
Postgraduation level				
	Doctorate (direct access)	32 (55)	39 (63)	0.407
	Master’s	16 (28)	10 (16)	
	Master’s and doctorate	10 (17)	10 (17)	
Postgraduation scholarship				
	Yes	13 (22)	13 (21)	1
	No	45 (78)	49 (79)	
Research project funding				
	Yes	6 (10)	7 (11)	1
	No	52 (90)	55 (89)	
Motivations to pursue postgraduate studies				
	Personal growth	35 (60)	43 (69)	0.341
	Professional growth	52 (90)	56 (90)	1
	Increase income levels	9 (16)	5 (08)	0.260
	Obtain a postgraduate scholarship	1 (02)	2 (03)	1
	Affinity for the research field	34 (59)	31 (50)	0.365
	Change of professional area	2 (03)	0 (00)	0.232
	Quality of the institution’s education	35 (60)	30 (48)	0.204
	Good reputation of the faculty	26 (45)	22 (35)	0.353
	Become a researcher	19 (33)	16 (26)	0.428
	Become a teacher/professor	24 (41)	27 (44)	0.855
	Pass public exams	4 (07)	7 (11)	0.532
	Opportunities in the job market	15 (26)	16 (26)	1
After completing postgraduate studies, which aspects were positively impacted?				
	Personal growth	46 (79)	48 (77)	0.828
	Professional growth	49 (84)	51 (82)	0.810
	Increased income levels	16 (28)	12 (19)	0.388
	Changed professional area	3 (05)	0 (00)	0.110
	Became a researcher	12 (21)	11 (18)	0.817
	Became a teacher/professor	17 (29)	19 (31)	1
	Passed public exams	10 (17)	7 (11)	0.435
	Had new job opportunities	22 (38)	13 (21)	0.047
Postdoctoral studies				
	Yes	8 (14)	12 (19)	0.469
	No	50 (86)	50 (81)	
Academic title (Associate Professor)				
	Yes	0 (00)	9 (15)	0.003
	No	58 (100)	53 (85)	
Is your workplace in São Paulo, the capital?				
	Yes	29 (50)	46 (74)	0.008
	No	29 (50)	16 (26)	
Work in their city of origin				
	Yes	30 (52)	34 (55)	0.855
	No	28 (48)	28 (45)	
Predominant field of work				
	Healthcare/Clinical practice	48 (83)	58 (94)	0.252
	Research	1 (02)	1 (02)	
	Teaching	8 (14)	3 (05)	
	Management	1 (02)	0 (00)	
Institution of work				
	Public and private	28 (48)	32 (52)	0.725
	Only private	16 (28)	17 (27)	
	Only public	14 (24)	12 (19)	
	Not applicable	0 (00)	1 (02)	
Academic activities				
	Yes	42 (72)	50 (81)	0.388
	No	16 (28)	12 (19)	
Research activities				
	Yes	29 (50)	42 (68)	0.063
	No	29 (50)	20 (32)	
Number of papers published				
	None	1 (02)	0 (00)	0.051
	1 to 5	22 (38)	11 (18)	
	6 to 20	24 (41)	22 (35)	
	21 to 50	7 (12)	15 (24)	
	51 to 100	4 (07)	8 (13)	
	More than 100	0 (00)	6 (10)	
Editorial board member				
	Yes	6 (10)	12 (19)	0.206
	No	52 (90)	50 (81)	
Leadership positions				
	Yes	31 (53)	32 (52)	0.857
	No	27 (47)	30 (48)	
Salary range				
	BRL 3.001 to BRL 10.000	5 (09)	0 (00)	0.004
	BRL 10.001 to BRL 20.000	10 (17)	3 (05)	
	More than BRL 20.000	36 (62)	45 (73)	
	Prefer not to disclose	7 (12)	14 (23)	
Digital platforms				
	ORCID	48 (83)	54 (87)	0.612
	LinkedIn	37 (64)	33 (53)	0.270
	Research Gate	24 (41)	32 (52)	0.278
	Google Scholar	15 (26)	29 (47)	0.023
	None	4 (07)	6 (10)	0.745

## DISCUSSION

 The postgraduate program evaluated in this study reflects the long-standing academic tradition of FMUSP, an institution that has played a central role in shaping medical and surgical education in Brazil^9^. With a response rate exceeding 50%, this study captured a broad spectrum of experiences and career outcomes among graduates over more than a decade. By surveying alumni from diverse backgrounds, our analysis provides a comprehensive overview of how postgraduate training has influenced personal and professional trajectories, while highlighting disparities and systemic challenges. 

 A key finding of this study is the substantial impact of postgraduate training on career development. More than half of the alumni considered the program fundamental to their professional trajectories, and an additional 37% reported a strong, though not decisive, impact. Importantly, 74% would certainly pursue postgraduate education again, underscoring the enduring value of this educational pathway. These findings are consistent with prior studies showing that academic degree training enhances career progression, academic productivity, and professional recognition in health sciences^4,6,14^. 

 Near gender parity was observed in the cohort (53.5% male vs. 46.5% female), a distribution that contrasts with the historical male predominance in surgical and academic careers. This aligns with broader national trends: women have been the majority of newly registered physicians in Brazil since 2009 and are soon expected to outnumber men among active physicians^12^. However, the predominance of male surgeons persists, reflecting well-documented gender disparities in the surgical field worldwide. 

 Of note, 33.7% of alumni completed only a master’s degree without advancing to doctoral studies. This reflects a structural challenge of the Brazilian postgraduate system, in which the sequential master’s-to-doctorate trajectory may discourage continuation. To mitigate this, the department has encouraged direct-entry doctoral admission, aiming to reduce barriers and maintain academic engagement after the master’s degree. 

 Strong contrasts were identified between physicians and non-physicians. Physicians were older, more likely to graduate from public universities, and had a higher prevalence of direct-entry doctoral degrees. They reported higher academic involvement, publication output, leadership roles, and income. Non-physicians were predominantly women, more frequently received scholarships and project funding, and more often reported career change as a meaningful impact. These findings highlight the multifaceted purposes of postgraduate programs, serving both for specialization and career redirection. 

 Differences were also observed among physicians based on surgical or clinical training. Surgeons were predominantly male, more concentrated in São Paulo, and more likely to hold senior academic titles. Clinicians reported more new job opportunities after postgraduate studies. These distinctions may reflect traditional gender imbalances in surgery, the concentration of surgical services in large cities, and the greater academic competitiveness of surgical fields. 

 The geographic distribution analysis highlights a persistent challenge: the concentration of highly trained professionals in São Paulo and Southeastern Brazil. Although alumni came from all Brazilian regions, most continued to work in São Paulo city, reflecting the clustering of tertiary hospitals, research institutions, and better professional opportunities. This is consistent with findings by Pierantoni et al.^11^, who demonstrated that disparities in healthcare infrastructure and career prospects strongly favor retention in major urban centers. Reducing this imbalance will require coordinated policies that expand regional academic capacity, strengthen funding mechanisms, and offer meaningful incentives for professionals to work in underserved regions^11,12^. 

 Financial aspects also emerged as relevant. Fewer than 35% of alumni received scholarships, primarily from CAPES, FAPESP, and CNPq, and only 16% reported project funding. These findings highlight ongoing challenges in sustaining postgraduate training in Brazil, particularly given the long duration of studies and the high cost of living in major urban centers. 

 This study has limitations. As a cross-sectional survey, it relies on self-reported data, which may be subject to recall bias. Being restricted to a single institution limits the generalizability of findings. Furthermore, non-respondents may include alumni who are less satisfied with their postgraduate experience, potentially skewing results toward more favorable perceptions. Nevertheless, the response rate and sample size strengthen the robustness of our analysis. 

 Taken together, these findings provide an unprecedented and comprehensive view of the academic and professional development of alumni from one of Brazil’s most traditional postgraduate programs in gastroenterology. By offering detailed insights into career paths, academic engagement, geographic distribution, and perceived impact, this study fills an important knowledge gap in the national literature and generates evidence that can guide program leadership, institutional planning, and policy discussions within CAPES and beyond. The results also highlight the strategic role of interdisciplinary postgraduate training in strengthening the healthcare and scientific workforce. As such, the study not only documents the trajectories of graduates but also helps shape future directions for postgraduate education in Brazil (Table 2). 

## CONCLUSIONS

 This survey of alumni from a postgraduate program in Sciences in Gastroenterology demonstrates that postgraduate studies have a strong impact on both personal and professional development. Most alumni reported career and academic growth, and the majority would certainly enroll again. Differences between physicians and non-physicians, and between surgical and clinical training, highlight the diverse ways postgraduate education shapes careers. At the same time, limited research funding and the concentration of professionals in large urban centers remain persistent challenges. These findings reinforce the pivotal role of postgraduate education in advancing health sciences while pointing to opportunities for greater equity and improved regional distribution of qualified professionals. 

## Data Availability

The datasets generated and/or analyzed during the current study are available from the corresponding author upon reasonable request.
